# ADVERSE CHILDHOOD EXPERIENCES REPORTED BY CUSTODIAL GRANDMOTHERS AND THEIR ADOLESCENT GRANDCHILDREN

**DOI:** 10.1093/geroni/igz038.1045

**Published:** 2019-11-08

**Authors:** Gregory C Smith, Frank J Infurna, Britney A Webster, Megan L Dolbin-MacNab, Max Crowley, Carol M Musil

**Affiliations:** 1 Kent State University, Kent, Ohio, United States; 2 Arizona State University, Tempe, Arizona, United States; 3 Virginia Tech, Blacksburg, Virginia, United States; 4 Pennsylvania State University, University Park, Pennsylvania, United States; 5 Case Western Reserve University, Cleveland, Ohio, United States

## Abstract

The Risky Family Model postulates that adverse childhood experiences (ACE) are likely to be encountered across generations within custodial grandfamilies which, in turn, may adversely impact their overall well-being. The present study is a pioneering attempt to examine the patterns of ACEs self-reported by custodial grandmothers (CGM) and adolescent grandchildren (AGC) from the same families, and how their total ACE scores correlate with key physical and mental health outcomes. A total of 129 CGM-ACG dyads recruited for a nationwide RCT study completed separately at baseline the 10-item ACE-CDC and 4 items from the ACE-IQ, as well as various standardized measures of physical and emotional well-being. The most frequent ACEs reported by AGC were loss of a parent (60.5%), verbal abuse (58.1%), bullying by peers (46.5%), and living with someone jailed (45.0%). The predominant ACEs for CGM were bullying by peers (48.8%), verbal abuse (48.1%), living with a mentally ill person (34.1%), being touched sexually (29.5%), and loss of parent (29.5%). Only 10.1% of ACG and 15.5% of CGM reported 0 ACEs, whereas 65.1 % of ACG and 59% of CGM reported > 3 ACEs. For ACG, total ACE scores correlated significantly with externalizing (r=.32) and internalizing (r=.30) difficulties, self-esteem (r= -.28), loneliness (r=.27), school problems (r=.24), and physical health (r= -.26). For CGMs, anxiety (r=.23) and depression (r=.19) only were correlated significantly with total ACEs. We conclude that although both CGM and ACG reported alarmingly high levels of ACEs, different patterns and correlates exist between the generations. [Funded by R01AG054571]

